# Nonalcoholic or metabolic-associated fatty liver disease and colorectal polyps: evidence from meta-analysis and two-sample Mendelian randomization

**DOI:** 10.3389/fgene.2024.1422827

**Published:** 2024-08-09

**Authors:** Dong Zhai, Sumei Xu, Haoge Liu, Xiaojuan Tong

**Affiliations:** ^1^ The Third Affiliated Hospital of Zhejiang Chinese Medical University (Zhongshan Hospital of Zhejiang Province), Hangzhou, China; ^2^ The First Affiliated Hospital of Zhejiang Chinese Medical University (Zhejiang Provincial Hospital of Traditional Chinese Medicine), Hangzhou, China

**Keywords:** nonalcoholic fatty liver disease, metabolic-associated fatty liver disease, colorectal polyps, meta-analysis, Mendelian randomization

## Abstract

**Introduction:**

Nonalcoholic or metabolism-associated fatty liver disease (NAFLD or MAFLD) and colorectal polyps are chronic conditions strongly linked to lifestyle factors. However, the precise causal link between NAFLD or MAFLD and the development of colorectal polyps is not yet fully understood. This study aimed to evaluate the association between NAFLD or MAFLD and the risk of colorectal polyps based on a meta-analysis and two-sample Mendelian randomization (MR) analyses.

**Methods:**

PubMed, Embase, Cochrane Library databases were searched for eligible studies to be included in the meta-analysis. We conducted a thorough search of the PubMed, Embase, and Cochrane Library databases to identify eligible studies prior to 22 March 2024. Subgroup analyses were performed based on sex, age, and geographical region. Causality between NAFLD/MAFLD and colorectal polyps was explored by using two-sample Mendelian randomization (MR) analyses.

**Results:**

Based on an analysis of 17 studies encompassed within this meta-analysis, a significant correlation was identified between the presence of NAFLD/MAFLD and elevated incidence of colorectal polyps (NAFLD: OR = 1.57, 95% CI: 1.43–1.73, I^2^ = 38%, *p* = 0.06; MAFLD: OR = 1.67, 95% CI: 1.40–2.00, I^2^ = 77%, *p* = 0.002). However, current evidence does not support a causal relationship between NAFLD/MAFLD and the prevalence of colorectal polyps (OR = 0.9998315, 95% CI: 0.9987566–1.000907, *P* = 0.7587638).

**Conclusion:**

NAFLD/MAFLD demonstrated a significant positive correlation with an elevated risk of developing colorectal polyps. However, the MR analysis suggested that no causal relationship existed between NAFLD/MAFLD and colorectal polyps. Therefore, further research is required to identify the underlying mechanism of causal link between these diseases.

## 1 Introduction

Colorectal cancer (CRC) has ascended to become the second most prevalent form of cancer globally (Edwards et al., 2014; Siegel et al., 2023). Furthermore, the incidence of CRC is witnessing a marked escalation, particular among individuals under the age of 55. The majority of CRC cases are known to originate from colorectal polyps, particularly adenomatous polyps that are widely acknowledged as the precursors to CRC. Colonoscopy is crucial diagnostic tool for detecting polyp progression in the general population. Thanks to the implementation of enhanced colonoscopy screening protocols, there has been a significant reduction in the overall incidence rates of CRC. However, some young patients with CRC do not have a family history of cancer (Dozois et al., 2008; Stoffel et al., 2018). Therefore, other modifiable risk factors of CRC and colorectal polyps need to be identified to diagnose and prevent disease progression.

Nonalcoholic fatty liver disease (NAFLD) has become the most prevalent form of chronic liver disease worldwide (Rinella and Sanyal, 2016; Zhou et al., 2020), affecting approximately 25% of the global population (Younossi et al., 2016; Younossi et al., 2018), and it will become the main cause of end-stage liver disease in the future (Dozois et al., 2008). NAFLD is not only associated with a high frequency of metabolic comorbidities (Cui et al., 2023), but also with high mortality rates in cardiovascular disease and malignancies. The establishment of the term “metabolically associated fatty liver disease” (MAFLD) was aimed to better define NAFLD in patients with metabolic syndrome (Lonardo et al., 2019). Patients diagnosed with NAFLD are reclassified as having MAFLD if they exhibit one or more of the specified coexisting conditions (Agustanti et al., 2023): 1) overweight or obesity, 2) type 2 diabetes (2-DM), or 3) evidence of metabolic abnormalities. The estimated incidence rate of MAFLD in the global adult population is approximately 25% (Le et al., 2019), and the rise in incidence among young individuals has become a matter of concern. The prevalence of MAFLD in lean adults was approximately 4.1%–34% (Eslam et al., 2022).

Both NAFLD/MAFLD and colorectal polyps are intimately linked to metabolic syndrome, sharing a multitude of risk factors, such as obesity, insulin resistance, diabetes, hyperlipemia and cardiovascular disease. Extensive research has demonstrated that individuals with NAFLD face an elevated risk of colorectal polyp development. The chronic inflammatory milieu characteristic of NAFLD is believed to foster the progression of these polyps, potentially paving the way for the advancement to colorectal cancer. Li et al. have provided evidence that the incidence of colorectal polyps is higher among patients diagnosed with NAFLD, as compared to those without this condition (Li et al., 2019). Chang et al. also suggested that MAFLD is associated with a higher incidence of adenomas across both younger and older individuals (Chang et al., 2023). The robust correlation between hepatic steatosis and insulin resistance potentially illuminates the underlying biological nexus that connects NAFLD/MAFLD with the development of colorectal polyps, suggesting a shared metabolic pathway. Insulin resistance is not only a typical clinical feature of NAFLD, but also a dependent metabolic abnormality in patients with colorectal polyps. Insulin signaling is involved in the pathogenesis of colorectal polyps. Furthermore, NAFLD/MAFLD is characterized by a state of persistent chronic inflammation, which is thought to be a driving force in the pathogenesis of colorectal polyps. The involvement of diverse cytokines, including interleukin-8 (IL-8), IL-6, and tumor necrosis factor-α (TNF-α), suggests a complex inflammatory milieu that may significantly impact the initiation and progression of these polyps. However, several clinical observational studies have demonstrated controversial results (Touzin et al., 2011; Mahamid et al., 2017; Yang et al., 2023). In addition, MAFLD is a novel diagnostic paradigm, encompassing individuals with metabolic syndrome who also exhibit characteristics of NAFLD. Currently, there is still a lack of meta-analysis investigating the relationship between MAFLD and colorectal polyps. Furthermore, the causal linkage between NAFLD/MAFLD and colorectal polyps has yet to be elucidated. Consequently, it is deemed valuable to delve into the potential effects of NAFLD/MAFLD on the risk profile for colorectal polyp development.

Mendelian randomization (MR) is a method using genetic variation to examine the causal associations between exposure and outcomes. Employing the random allocation of alleles to reveal genetic variance, we can effectively sidestep the confounding factors typically present in observational research, thereby enabling a more accurate determination of causality. In recent years, a multitude of Mendelian randomization (MR) studies have come to the forefront, providing compelling clinical evidence and solidifying MR’s reputation as a robust and credible research approach for tackling complex clinical inquiries. Taking advantage of the latest pooled data for NAFLD/MAFLD and summary statistics for colorectal polyps derived from genome-wide association study (GWAS), we attempted to explore the causal association between NAFLD/MAFLD and colorectal polyps by utilizing a two-sample MR. We adopted a two-stage analytical approach to detect the potential interplay between NAFLD/MAFLD and colorectal polyps. First, we conducted a comprehensive meta-analysis to refine the prevalence estimates of colorectal polyps among individuals with NAFLD/MAFLD. Second, we proceeded with a MR analysis to ascertain the causality between NAFLD/MAFLD and the occurrence of colorectal polyps.

## 2 Methods

### 2.1 Meta-analysis

#### 2.1.1 Search strategy and eligibility criteria

In accordance with the PRISMA 2020 guidelines, we conducted a systematic search across PubMed, EMBASE, and the Cochrane Library to identify studies published prior to 22 March 2024. The search keywords were as follows: (Non-alcoholic Fatty Liver Disease OR Non alcoholic Fatty Liver Disease OR NAFLD OR NAFL OR NASH OR Nonalcoholic Fatty Liver Disease OR Nonalcoholic Fatty Liver OR Nonalcoholic Steatohepatitis OR Nonalcoholic Steatohepatitis OR MAFLD OR Metabolic Associated Fatty Liver Disease OR metabolic dysfunction-associated fatty liver disease) AND (Polyps OR Polyp OR Adenomatous Polyps OR Intestinal Polyps OR Colonic Polyps OR Colorectal Neoplasms OR Colorectal Tumor OR Colorectal Cancer OR Colorectal Carcinoma OR colorectal polyps OR colorectal polyps OR colorectal adenomas). The studies were included based on the following criteria: 1) case-control, cross-sectional, and cohort designs; 2) published in the English language; 3) the association of NAFLD/MAFLD and colorectal polyps was evaluated; and, 4) results should include the odds ratio (OR), risk ratio (RR), and hazard ratio (HR) with 95% confidence interval (CI), or sufficient data to calculate them. The exclusion criteria were as follows: 1) duplicate studies; 2) animal experiments, meta-analyses, reviews, conference proceedings, case reports, and guidelines; 3) studies with obvious defects and significant biases; and, 4) studies without useful data. The meta-analysis was registered in PROSPERO (CRD42024528397).

#### 2.1.2 Data extraction

Two investigators (Xiaojuan Tong and Dong Zhai) independently extracted information from the eligible studies based on predefined criteria. Any discrepancies that arose during the screening process were resolved by collaborative consensus, with a third reviewer, Sumei Xu. The extracted data encompassed a comprehensive set of variables, including the name of the first author, publication year, country, sex, age, incidence of polyps in the NAFLD or MAFLD group, incidence of polyps in the control group, study type, adjusted confounding factors, and type of colorectal polyps. The Newcastle-Ottawa scale (NOS) was used to evaluate the quality and risk of bias of the included studies. We identified studies with ≥7 as high-quality studies (Table 1).

**TABLE 1:** Quality assessment of included studies via the Newcastle-Ottawa scale (NOS).

Study	Selection				Comparability control for important factor	Exposure			Total score
	Adequate case definition	Representativeness Of the cases	Selection of controls	Definition of controls		Ascertainment of exposure	Same method of ascertainment for cases and controls	Non-response rate	
Yang et al. (2023)	1	1	1	0	2	1	1	0	7
Touzin et al. (2011)	1	1	1	1	2	1	1	0	8
Mahamid et al. (2017)	1	1	1	1	2	1	1	0	8
Lesmana et al. (2020)	1	1	1	1	1	1	1	0	7
Hwang et al. (2010)	1	1	1	0	2	1	1	0	7
Cho et al. (2019)	1	1	1	1	2	1	1	0	8
Chen et al. (2017)	1	1	1	1	2	1	1	0	8
Chao et al. (2020)	1	1	1	1	2	1	1	0	8
Blackett et al. (2020)	1	1	11	1	2	1	1	0	8
Bhatt et al. (2015)	1	1	1	1	2	1	1	0	8
Seo et al. (2021)	1	1	1	1	2	1	1	0	8
Li et al. (2019)	1	1	1	1	2	1	1	0	8
Huang et al. (2013)	1	1	1	1	2	1	1	0	8
Fukunaga et al. (2021)	1	1	1	1	1	1	1	0	7
Chang et al. (2023)	1	1	1	1	2	1	1	0	8
Stadlmayr et al. 2011	1	1	1	1	2	1	1	0	8
Fliss-Isakov et al. (2011)	1	1	1	1	2	1	1	0	8

#### 2.1.3 Statistical analysis

The data were analyzed and visualized by using RevMan5.3 statistical software. Statistical significance was ascertained using the pooled *P*-value as the metric, with a threshold of *P* < 0.05 established to denote statistical significance. The comparative prevalence of colorectal polyps between patients with NAFLD or MAFLD and the control group was assessed through the computation of the odds ratio (OR) and 95% confidence interval (CI). To assess the degree of heterogeneity within the pooled studies, we performed the Cochran Q-test and calculated the I2 statistic. A random effects model was applied when *I* ^2^ > 50% and *P* < 0.05. Conversely, a fixed effects model was utilized. Subgroup analysis was performed to detect potential heterogeneity. A sensitivity analysis was employed by removing one study at a time to evaluate the stability of the meta-analysis results. Funnel plots was used to assess publication bias.

### 2.2 Mendelian randomization analysis

#### 2.2.1 Data source

Two-sample Mendelian randomization (MR) analysis was used to detect the causal association between NAFLD/MAFLD and colorectal polyps based on a publicly available large-sample genome-wide association study (GWAS) database. GWAS summary information on NAFLD or MAFLD and colorectal polyps was acquired from the IEU Open GWAS Database (http://gwas.mrcieu.ac.uk/). Summary data for NAFLD/MAFLD was obtained from EBI database and colorectal polyps GWAS from the UK Biobank. The dataset IDs for NAFLD/MAFLD and colorectal polyps were as follows: ebi-a-GCST90054782 with 3,77,998 individuals (4,761 European cases and 3,73,227 European controls) and ukb-b-14210 with 4,62,933 individuals (1,391 European cases and 4,61,542 European controls).

#### 2.2.2 Selection of SNP

In order to ensure the accuracy and reliability of the study results, we adhered to stringent criteria for the selection of single nucleotide polymorphisms (SNPs). SNPs associated with NAFLD/MAFLD were selected under the genome-wide significance threshold (*P* < 5 × 10^−8^). PLINK clumping algorithm (r2 = 0.001, clustering distance = 10,000 kb) was used to cluster SNPS to ensure the independence of each SNP.

#### 2.2.3 Assumption of MR analysis

There were three core assumptions of MR analysis to minimize bias in the results:1. Relevance hypothesis: The extracted instrumental variables (IVs) must be directly related to exposure. 2. Independence assumption: IVs must be independent of any confounding factors in the exposure outcome association. 3. Exclusion of the limiting hypothesis: IVs must influence results only through exposure. F statistic, whose formula is F = Beta^2^/SE^2^ was utilized to evaluated the strength of the relationship between IVs and exposure, and to eliminate weak IVs. Here, Beta and SE represent the estimated effect and standard error of the allele on exposure, respectively. When IVs with F statistics less than 10, we usually consider the IVs as weak, and delete them to avoid potential genetic confusion or measurement errors.

#### 2.2.4 Statistical analysis

R4.3.2 was used for MR analysis in the present study. The MR methods used in this study to evaluate the causal effect of NAFLD/MAFLD on colorectal polyps were as follows: weighted median regression, inverse variance weighting (IVW), Mendelian randomization Egger (MR Egger), simple mode, and weighted mode. Sensitivity analyses were conducted using a comprehensive set of methods, such as Cochran’s Q statistic, MR pleiotropy residual sum and outlier (MR-PRESSO), and leave-one-out analyses. Cochran’s Q statistic was used to examine the heterogeneity among SNPs. Outlier SNPs were identified using the MR-PRESSO package and removed to eliminate horizontal pleiotropy. We performed a leave-one-out analysis by deleting SNPs one by one to assess the consistency and robustness of the results, and R^2^ is the extent to which IVs explain exposure.

## 3 Results

### 3.1 Meta-analysis of observational studies

#### 3.1.1 Characteristics and quality assessment of the included studies

**FIGURE 1:**
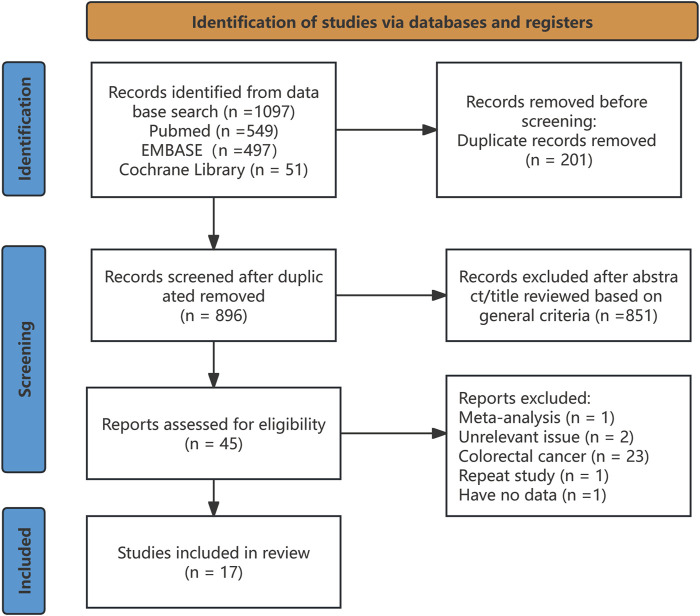
Flowchart of the literature search.

**FIGURE 2:**
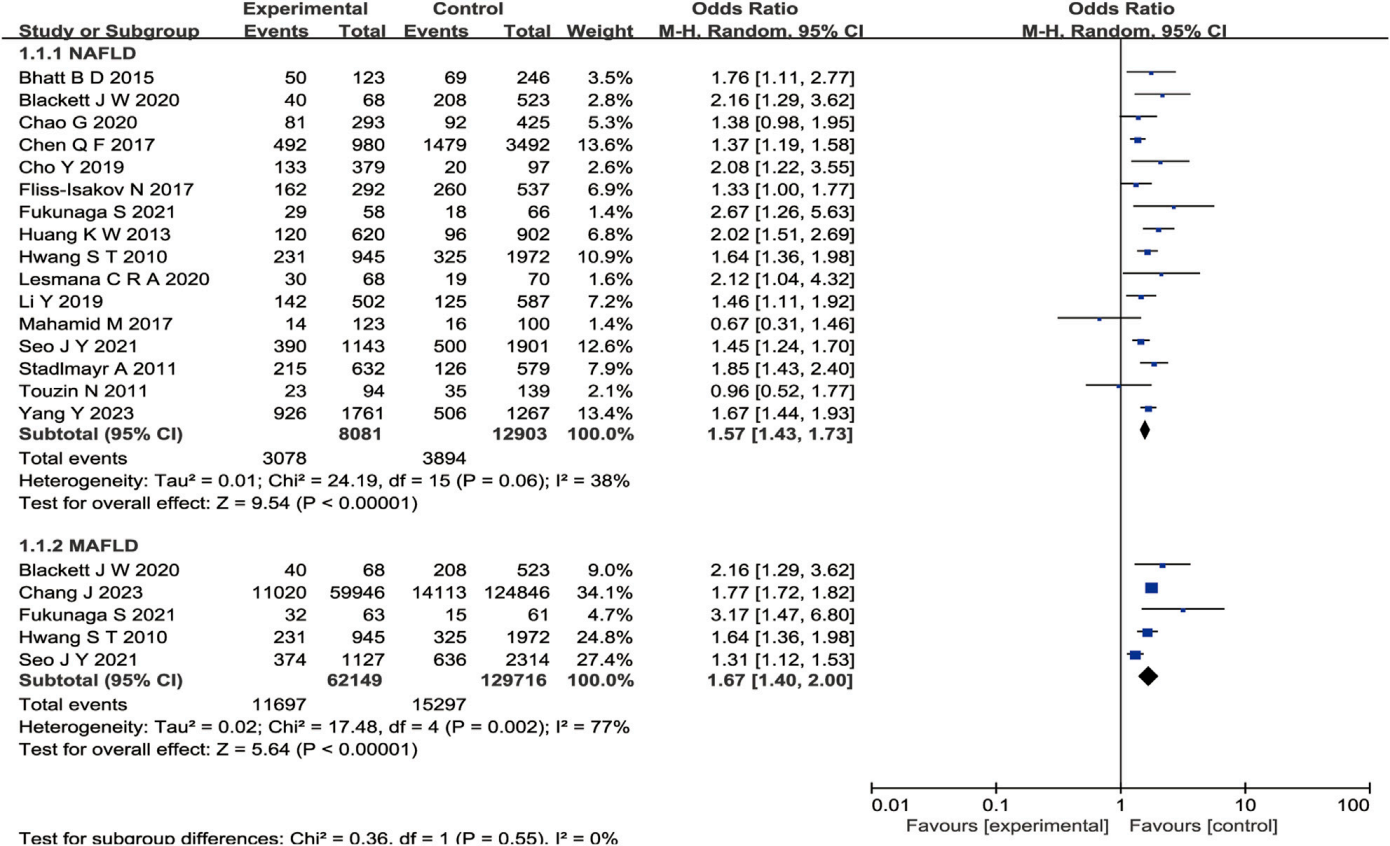
Forest plot of comparison. The effect of NAFLD *versus* MAFLD on the prevalence of colorectal polyps.

We obtained 17 studies (Hwang et al., 2010; Stadlmayr et al., 2011; Huang et al., 2013; Bhatt et al., 2015; Chen et al., 2017; Fliss-Isakov et al., 2017; Zelber-Sagi et al., 2018; Cho et al., 2019; Li et al., 2019; Blackett et al., 2020; Chao et al., 2020; Lesmana et al., 2020; Fukunaga et al., 2021; Seo et al., 2021; Sung et al., 2021; Chang et al., 2023; Llovet et al., 2023) based on the inclusion and exclusion criteria. The detailed selection procedure is summarized in Figure 1. The descriptive characteristics of each study are presented in Table 2. There were 12 retrospective studies (two retrospective, four retrospective cross-sectional, and six retrospective cohort studies); two, cross-sectional studies; two, cohort studies; and, one, case–control study, which were published between 2010 and 2023. The forest plot indicates a statistically significant association between NAFLD/MAFLD and an elevated risk of colorectal polyps (Figure 2). The pooled OR was 1.57 with *p* < 0.01 (95% CI 1.43–1.73) for NAFLD and 1.67 with *p* < 0.01 (95% CI 1.40–2.00) for MAFLD. Statistical heterogeneity was low among the selected studies on NAFLD (*p* = 0.06, I^2^ = 38%), and high among studies based on MAFLD (*p* = 0.002, I^2^ = 77%).

**TABLE 2:** Characteristics of studies on between NAFLD/MAFLD and the risk of colorectal polyps.

Author (year)	Country	Sex (M/F)	Age (year)	Polyps in NAFLD/MAFLD	Polyps in control	Study type	Adjusted confounding factors	Type of polyps
Yang et al. (2023)	China	1728/1300	54.4 ± 14.0	NAFLD (926)	Non-NAFLD (506)	Retrospective cross-sectional	age, sex, TG, TC, LDL-C and HDL-C	Adenomatous
Touzin et al. (2011)	United States	120/113	54.7 ± 6.0	NAFLD (23)	Non-NAFLD (35)	Retrospective cohort observational	race, BMI, and family history	Adenomas
Mahamid et al. (2017)	Israel	115/108	41.1 ± 12.5	NAFLD (14)	Non-NAFLD (16)	Retrospective cohort observational	gender, age, C-reactive protein and smoking	hyperplastic polyp
Lesmana et al. (2020)	Korea	72/66	56.8 ± 15.3	NAFLD (30)	Non-NAFLD (19)	Retrospective	No adjusted	Adenoma
Hwang et al. (2010)	Korea	1911/2917	47.0 ± 9.0	NAFLD (231) MAFLD (67)	Non-NAFLD (325) Non-MAFLD (258)	Cross-sectional	age, gender, smoking, NAFLD, metabolic syndrome hypertension and diabetes	Adenomatous polyp
Cho et al. (2019)	China	230/246	55.9 ± 12.9	NAFLD (133)	Non-NAFLD (20)	Cohort	Age, Sex, Diabetes mellitus, Antidiabetic drug use, Hypertension, Antihypertensive drug use, Statin use, Smoking, hsCRP, HOMA‐IR, Lobular inflammation, Ballooning, Steatosis grade, Significant fibrosis, Histological spectrum of NAFLD	Low‐grade tubular adenoid Advanced colorectal neoplasm
Chen et al. (2017)	China	2430/1256	47.5 ± 10.6	NAFLD (492)	Non-NAFLD (1479)	Retrospective cross-sectional	gender, age, smoking, alcohol and MS.	Adenomatous polyps, Hyperplastic polyps
Chao et al. (2020)	China	469/249	49.4 ± 8.2	NAFLD (81)	Non-NAFLD (92)	Retrospective	No adjusted	Adenoma
Blackett et al. (2020)	United States	183/186	60.4 ± 8.9	NAFLD (50) MAFLD (40)	Non-NAFLD (69) Non-MAFLD (90)	Retrospective cross-sectional	Rates of hyper-lipidemia, diabetes, and obesity	Adenoma
Bhatt et al. (2015)	United States	398/193	59.8	NAFLD (40)	Non-NAFLD (208)	Retrospective cohort	age	Adenoma, Inflammatory, Hyperplastic
Seo et al. (2021)	Korea	2130/1311	52.4 ± 9.1	NAFLD (390) MAFLD (374)	Non-NAFLD (500) Non-MAFLD (636)	Retrospective cohort	age, sex, smoking, triglyceride level, HDL cholesterol level, hypertension, visceral fat area, diabetes, and body mass index	Adenoma
Li et al. (2019)	China	566/523	54.5 ± 0.6	NAFLD (142)	Non-NAFLD (125)	Retrospective cohort	sex, NAFLD, CAP, body mass index, triglyceride, aspartate aminotransferase, and fasting plasma glucose	Adenoma
Huang et al. (2013)	China	890/612	53.7 ± 9.7	NAFLD (120)	Non-NAFLD (96)	Retrospective cohort	Age, Body mass index, Male gender, Nonalcoholic fatty liver disease, Smoking, Hypertension, Diabetes mellitus	Adenoma
Fukunaga et al. (2021)	Japan	100/24	59 ± 11.1	NAFLD (29) MAFLD (32)	Non-NAFLD (18)	Retrospective cross-sectional	No adjusted	Adenoma
Chang et al. (2023)	Korean	55,788/92,762	42.6 ± 9.4	MAFLD (11,020)	Non-MAFLD (14,113)	cross- sectional	age, sex, centre, year of screening, smoking status, alcohol intake, educational level, a history of cardiovascular disease, and a family history of colorectal cancer	Low- risk adenoma, or high- risk adenoma
Stadlmayr et al. (2011)	Austria	603/608	60.88 ± 9.98	NAFLD (215)	Non-NAFLD (126)	cohort	Sex, age, body mass index, Liver steatosis, Glucose intolerance	Tubular adenoma, advanced adenoma or carcinoma
Fliss-Isakov et al. (2017)	Israel	416/412	58.4 ± 6 6.6	NAFLD (162)	Non-NAFLD (260)	case-control	age, gender, BMI, low socioeconomic status, Ashkenazi origin, family history of colorectal malignancy, smoking (ever), alcohol consumption, physical inactivity, and use of statins, aspirin, and nonsteroidal anti-inflammatory drugs	Hyperplastic polyps, Serrated adenomas, Adenomas

#### 3.1.2 Subgroup analysis

Subgroup analyses were performed with stratification according to gender, age, and geographic region for studies on NAFLD and by age and diagnostic differences for those on MAFLD (Table 3). A subgroup analysis conducted by geographic region find a positive association between NAFLD and colorectal polyps (Asian: OR = 1.53, 95% CI: 1.43–1.64; non-Asian: OR = 1.75, 95% CI: 1.45–2.13). There was no statistically significant heterogeneity among the studies (Asian, *P* = 0.08 and non-Asian, *P* = 0.21). On stratifying by age, the pooled ORs of studies were 1.41 (95% CI: 1.17–1.69, *P* = 0.11) for those aged <50 years, and 1.64 (95% CI: 1.48–1.82, *P* = 0.19) for those aged ≥50 years. On stratifying by sex, there was statistically significant heterogeneity among men but not among women. The pooled ORs of studies were 1.63 (95% CI: 1.11–2.38, *P* = 0.0003) for men and 1.39 (95% CI: 1.15–1.67, *P* = 0.43) for women. Regarding MAFLD, there is a statistical difference in the age subgroup analysis (age <50 years: OR = 2.06, 95% CI: 1.47–2.88, *P* = 0.04 and age ≥50 years: OR = 1.47, 95% CI: 1.35–1.59, *P* = 0.29). However, statistically significant heterogeneity was detected between diagnostic differences (NAFLD–MAFLD: OR = 1.70, 95% CI: 1.42–2.03, *P* = 0.32, I^2^ = 0 and MAFLD: OR = 1.75, 95% CI: 1.71–1.80, *P* = 0.0003, I^2^ = 88%). Furthermore, the subgroup analysis failed to pinpoint the source of heterogeneity.

**TABLE 3:** Subgroup analysis of the incidence of colorectal polyps with NAFLD/MAFLD.

Subgroup	Study included	OR (95%CI)	P^(interaction)^
NAFLD			
Gender			0.46
Male	3	1.63 (1.11–2.38)	
Female	3	1.39 (1.15–1.67)	
Age			0.15
<50 years	4	1.41 (1.17–1.69)	
≥50 years	10	1.64 (1.48–1.82)	
Region			0.19
Asia	12	1.53 (1.43–1.64)	
Non-Asia	4	1.75 (1.45–2.13)	
MAFLD			
Age			0.05
<50 years	2	2.06 (1.47–2.88)	
≥50 years	3	1.47 (1.35–1.59)	
Diagnosis deference			0.73
NAFLD-MAFLD	2	1.70 (1.42–2.03)	
MAFLD	3	1.75 (1.71–1.80)	

NAFLD: nonalcoholic fatty liver disease; MAFLD: Metabolic-Associated Fatty Liver Disease; 2-DM: Type 2 diabetes mellitus.

**TABLE 4:** Associations of genetic instruments for NAFLD with colorectal polyps.

Instrumental SNP	Effect allele	Other allele	Exposure (NAFLD/MAFLD)			Outcome (colorectal polyps)		
			β	SE	*P*-value	β	SE	*P*-value
rs1260326	C	T	−0.136025	0.0208660	2.53630e-11	−7.25233e-05	0.000214625	0.7400000
rs17321515	G	A	−0.154093	0.0207828	1.81343e-13	−1.27993e-04	0.000210322	0.5400000
rs2642442	T	C	0.137690	0.0227583	7.67132e-10	−1.79940e-04	0.000225517	0.4200000
rs3747207	A	G	0.369714	0.0229366	6.74062e-60	2.23223e-04	0.000255524	0.3800000
rs429358	C	T	−0.199223	0.0304411	2.16920e-11	0 6.27212e-04	0.000291014	0.0309999
rs73001065	C	G	0.345021	0.0348771	1.08143e-24	−3.13664e-04	0.000378865	0.4100000

*P*-Value <5 × 10^−8^ for reporting genome-wide significance. EAF, effect allele frequency. SE, standard error; SNP, single nucleotide polymorphism. NAFLD, non-alcoholic fatty liver disease. MAFLD, Metabolic-Associated Fatty Liver Disease.

#### 3.1.3 Sensitivity analysis

**FIGURE 3:**
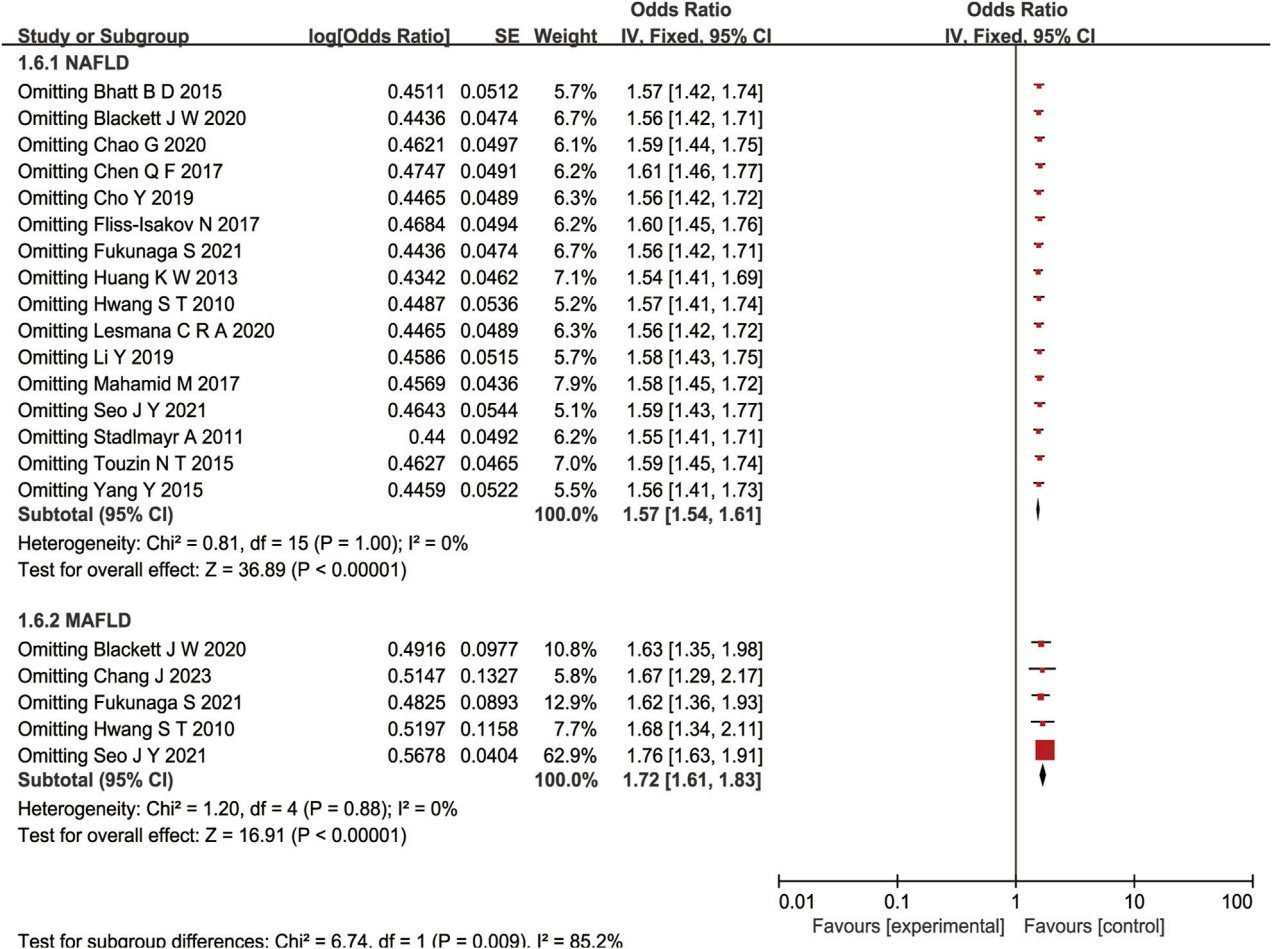
Forest plot of sensitivity analysis of association between NAFLD/MAFLD and risk of colorectal polyps.

**FIGURE 4:**
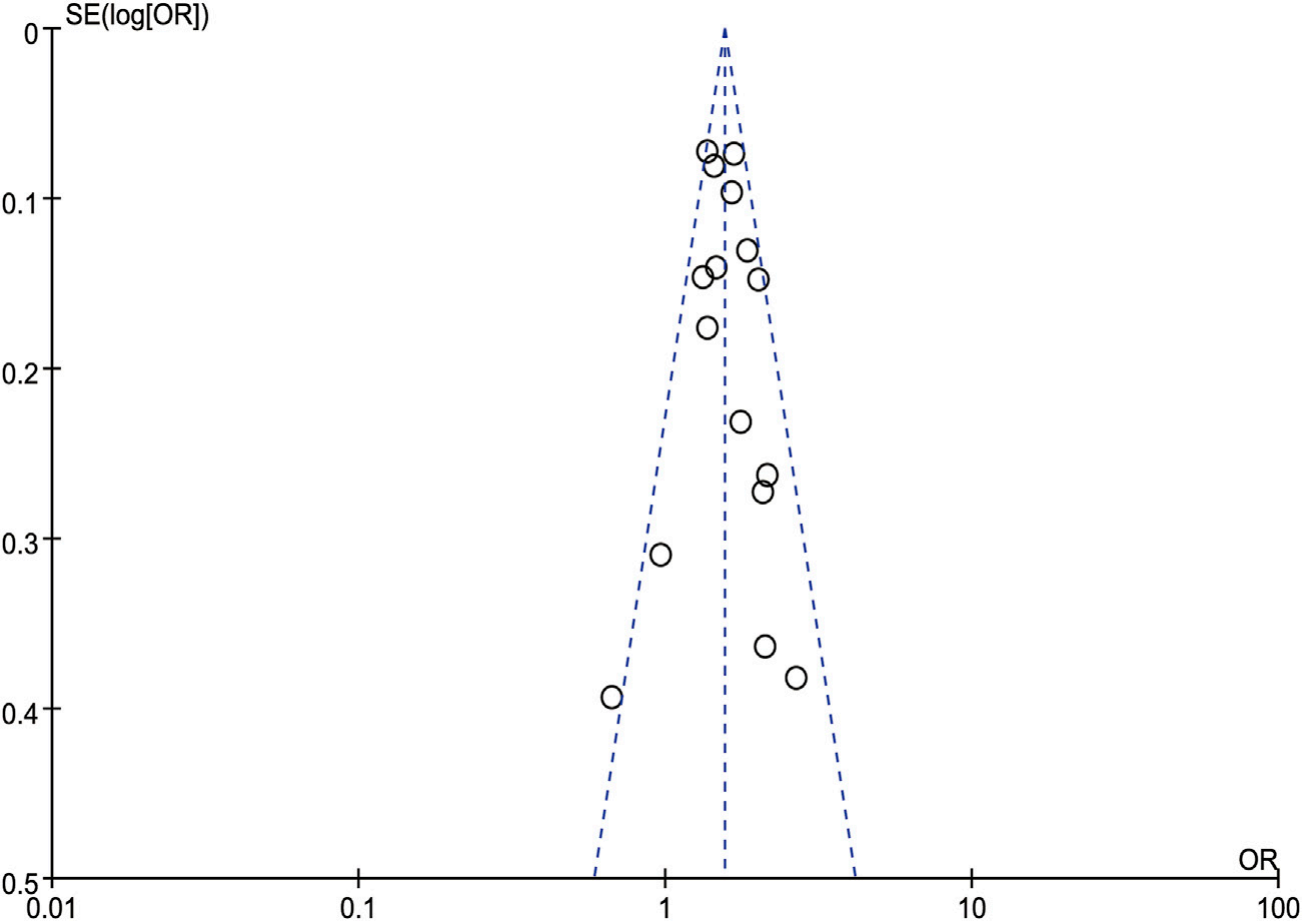
Funnel plot of meta-analysis for NAFLD.

A sensitivity analysis was performed by removing one study at a time and calculating the pooled ORs for the remaining studies. None of the studies changed the overall effect size (*P* > 0.05), indicating that the results were robust (Figure 3). A funnel plot was constructed by using Revman 5.3 software. The OR values of each study were used as the x-axis, and the reciprocal of the standard error of the OR values (SE [log OR]) was used as the y-axis. We have constructed the funnel plot for NAFLD, but not for MAFLD. The number of included studies for MAFLD were less than 10, hence the funnel plot was not suitable for publication bias evaluation. The results revealed an uneven distribution on both sides of the centerline (Figure 4), which may indicate a certain publication bias in the selected studies regarding the correlation between NAFLD and colorectal polyps.

### 3.2 MR analysis

#### 3.2.1 Characteristics of SNPs as instrumental variables

Six SNPs (rs2642442, rs1260326, rs17321515, rs73001065, rs429358, and rs3747207) were identified as IVs in NAFLD/MAFLD (Table 4). The calculated F-statistic for the selected SNPs was greater than 10 (F = 81.78161), indicating that the IVs had strong instruments. The value of R^2^ was 0.001512264.

#### 3.2.2 Genetical causal prediction of NAFLD on colorectal polyps

**FIGURE 5:**
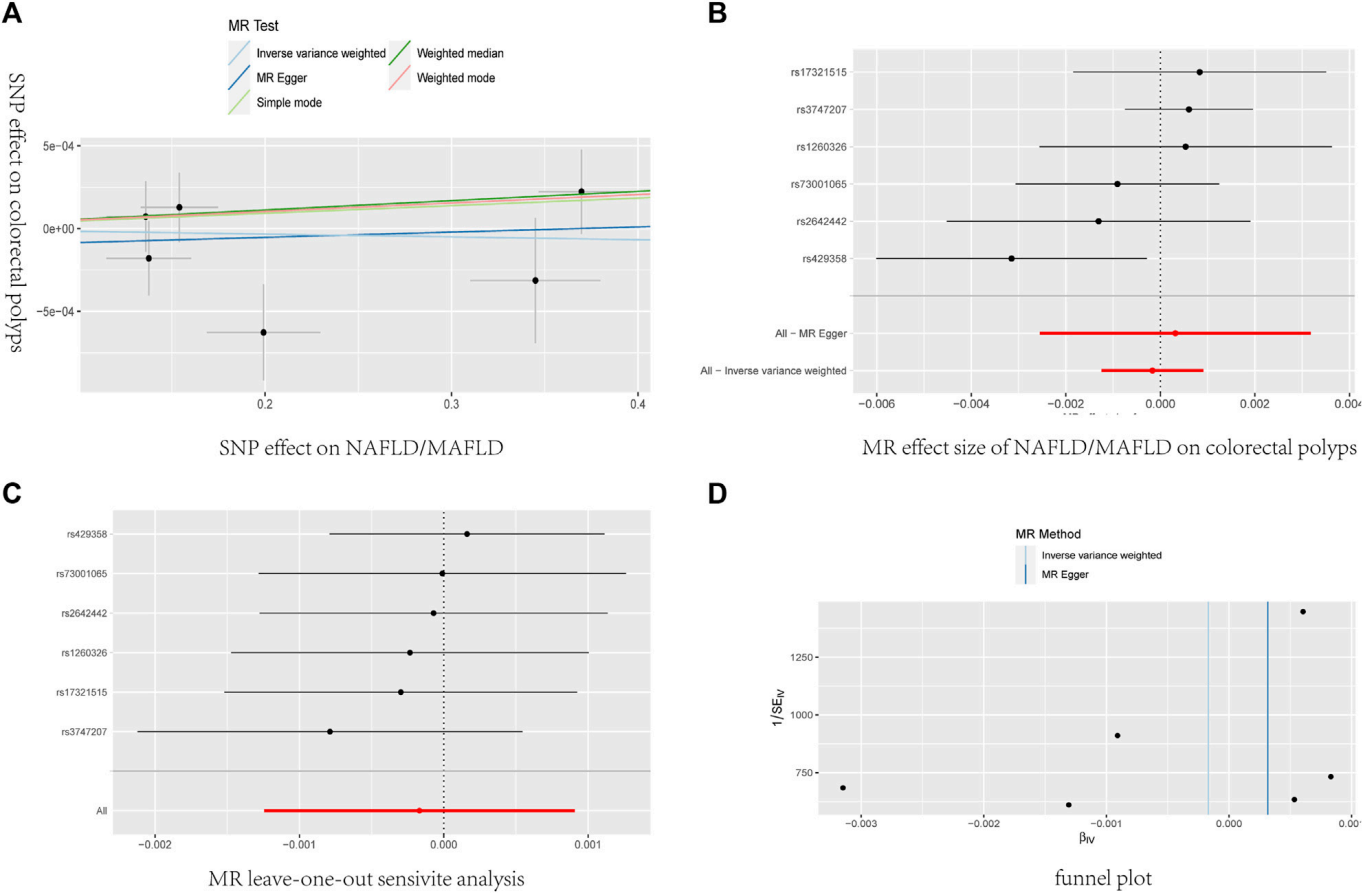
The causal effects of NAFLD/MAFLD on colorectal polyps in different MR methods. **(A)** Scatter plot. The slope of the line represents the magnitude of the causal relationship. **(B)** Forrest plot for overall heterogeneity of MR estimates for the effect of. **(C)** Leave-one-out analysis. **(D)** Funnel plot.

The main MR analysis method of IVW (OR = 0.9998, 95% CI: 0.9987–1.0009, *P* = 0.758) revealed no significant causal association between NAFLD/MAFLD and colorectal polyps. A consistent conclusion was also presented by the MR Egger method, weighted median, weighted mode and simple mode, as shown in Table 5; Figure 5.

**TABLE 5:** Causal association of NAFLD/MAFLD on colorectal polyps by MR analysis in different MR methods.

Exposure	Outcome	SNP(n)	method	OR	95% CI	P Val	Adjusted-P val
NAFLD/MAFLD	colorectal polyps	6	IVW	0.9998315	0.9987566–1.000907	0.758764	0.839398
		6	MR Egger	1.0003164	0.9974518–1.003189	0.839398	0.839398
		6	Weighted median	1.0005619	0.9993999–1.001725	0.343378	0.839398
		6	Weighted mode	1.0005137	0.9991743–1.001855	0.486265	0.839398
		6	Simple mode	1.0004606	0.9986291–1.002295	0.643193	0.839398

IVW: Cochran’s Q = 7.081, *P* = 0.214.

MR, Egger: Cochran’s Q = 6.854, *P* = 0.143.

MR-Egger intercept = −0.000116, *P* = 0.734.

MR-PRESSO, global test = 0.256.

#### 3.2.3 Sensitivity analysis

Heterogeneity was determined using Cochran’s Q statistic for IVW and the MR Egger method. No convincing evidence of heterogeneity was found by IVW (Q = 7.081, *P* = 0.214) or the MR–Egger method (Q = 6.854, *P*= 0.143). No potential SNP outliers were identified by the MR PRESSO test (*P* = 0.256). The MR–Egger intercept test revealed no horizontal pleiotropy (intercept = −0.000116; *P* = 0.734). Moreover, the leave-one-out sensitivity analysis revealed no significant outliers, demonstrating the stability of the MR analysis results (Table 5; Figure 5).

## 4 Discussion

The incidence of CRC in 2020 was more than 1.9 million new cases, and it has been recognized as the second leading cause of cancer-related deaths worldwide (Kasprzak, 2021). A high-fat and high-fructose diet, hyperlipidemia, obesity with a particular emphasis on visceral adiposity, and insufficient exercise are established risk factors for the development of colorectal polyps and NAFLD/MAFLD (Chen et al., 2020; Tong et al., 2021). Although NAFLD/MAFLD and colorectal polyps exhibit parallels in their pathogenic processes, the clinical interplay between these conditions remains a subject of debate. The Potential mechanisms link between NAFLD/MAFLD and the progression of colorectal polyps, particularly the roles of insulin resistance (IR) and chronic inflammation, have been widely reported.

To our knowledge, this is the first comprehensive study to evaluate the causal relationship between NAFLD/MAFLD and colorectal polyps using a meta-analysis and MR analysis. A number of prior meta-analyses have explored the relationship between NAFLD and colorectal polyps or adenomas, and some of these studies may encompass findings that intersect with the scope of our current investigation. However, our meta-analysis introduces a novel dimension by incorporating studies on MAFLD and colorectal polyps for the first time. In addition, this study represents the inaugural application of MR to evaluate the causal relationship between NAFLD and colorectal polyps. Our results suggest that NAFLD is significantly associated with an elevated risk of colorectal polyps development. The incidence of colorectal polyps in the NAFLD groups was 1.5 times higher than those in the non-NAFLD (*P* < 0.001) groups. These results are consistent with the findings of previous meta-analyses that focused on investigating the association between NAFLD and colorectal adenoma or tumor (Ding et al., 2015; Shen et al., 2014; Mantovani et al., 2018; Chen et al., 2019). Compared with the study by Chen et al. (Ding et al., 2015), our updated meta-analysis boasts a substantially larger sample size and a more exhaustive coverage of the literature. A total of 205,776 individuals from 17 studies were included in the meta-analysis. Furthermore, our result demonstrated a significant relationship between MAFLD and an increased risk of developing colorectal polyps (OR = 1.67, 95% CI: [1.40, 2.00], *P* = 0.002) for the first time. Compared with NAFLD, patients with MAFLD have a higher incidence of colorectal polyps (OR = 1.57, 95% CI: [1.43, 1.73] vs. OR = 1.67, 95% CI: [1.40, 2.00]). However, our MR analysis results did not support a causal role of NAFLD/MAFLD variants in the development of colorectal polyps.

High heterogeneity was not observed among the included studies on NAFLD. The Pooled analyses of age subgroups revealed a noteworthy pattern: compared to patients with NAFLD aged <50 years, patients aged >50 years were at a higher risk for colorectal polyps (OR = 1.41, 95% CI: 1.17–1.69 vs. OR = 1.64, 95% CI: 1.48–1.82). A similar situation was observed in the sex subgroup analysis. Compared with women diagnosed with NAFLD, male patients with NAFLD had a higher risk of developing colorectal polyps (OR = 1.63, 95% CI: 1.11–2.38 vs. OR = 1.39, 95% CI: 1.15–1.67). Our results are consistent with those of a large-scale study by Chang et al. (Chang et al., 2023). A regional subgroup analysis of Asian and non-Asian populations revealed a significant association between NAFLD and an increased risk of colorectal polyps; however, regional differences are not significant. Yang et al. () also showed no significant difference in the relationship between NAFLD and colorectal polyps in men and women. As for MAFLD, although there was great heterogeneity (*p* = 0.002, I 2 = 77%), the effect sizes were all on the same side of the nullify. Our findings are in concordance with the outcome of a large-scale study conducted by Chang et al. (OR = 1.67, 95% CI: 1.40–2.00). The results indicate a predisposition toward an increased prevalence of polyps among MAFLD patients. However, variations in effect sizes were observed. Previous studies have established a robust correlation between MAFLD and a multitude of factors, including age, sex, overweight or obesity, type 2 diabetes, hyperlipidemia, hypertension. These associations may underpin the observed heterogeneity. In our study, we conducted subgroup analyses focusing on age and diagnostic disparities. Regrettably, these analyses did not yield significant subgroup effects. The reasons may be as follows: 1) The sample size was not large enough for analysis because of the fewer studies were included. 2) There were no sufficient subgroup analyses to obtain the source of heterogeneity. Therefore, a comprehensive consideration of the multiple factors associated with MAFLD is essential for the improved design of future randomized controlled trials (RCT) and meta-analysis. Furthermore, several factors were identified that could potentially influence publication bias. Firstly, the sample size of several studies included in the literature was small. Secondly, the research population for these studies is predominantly concentrated in Asian countries. Finally, the assessment criteria for colorectal polyps exhibit a lack of uniformity across studies. These factors could be potential source of publication bias. Future RCTs necessitate a multicenter approach, high methodological quality, and substantial sample sizes to ensure robust verification of the findings.

Although the meta-analysis implied a significant correlation between NAFLD/MAFLD and the incidence of colorectal polyps, our findings do not provide conclusive evidence to establish a causal association between these diseases. There are several reasons for the inconsistent results between the meta-analysis and the MR analysis. First, the potential for residual confounding factors and the variability in covariate adjustment across studies could influence the estimation of the causal relationship between NAFLD/MAFLD and colorectal polyps. Incomplete elimination and adjustment for these factors may contribute to discrepancies in the findings. In our study, we identified that three of the included observational studies did not account for confounding factors, and a single study incorporated age as the sole adjustment variable (Table 2). This could have potential introduce a latent bias into our findings. Second, the subjectivity inherent in self-report or interview-based evaluation questionnaires, coupled with variability in the diagnostic techniques employed by different physicians for the detection of NAFLD/MAFLD and colorectal polyps may impart an element of uncertainty to the study outcomes.

The present study has several potential limitations. Firstly, subgroup analysis was conducted only by age, sex, and region because of insufficient original data in the selected studies. Moreover, only three of the included studies (Li et al., 2019) in the meta-analysis performed a stratified analysis based on sex. Therefore, we could not evaluate the influence of other confounding factors and subgroups on the association between NAFLD/MAFLD and colorectal polyps. Secondly, in the present meta-analysis, there was a notable predominance of Asian populations, with those from China, Korea, Japan constituting 89.7% of the sample. The inclusion of non-Asian studies was limited to populations from the USA and Austria. The participants enrolled in the MR analysis were only individuals of European ancestry. 3) The analysis relied on a select group of only six SNPs as IVs, which may have limited the accuracy of the causal inferences drawn between NAFLD and colorectal polyps. 4) While the strong F-statistic suggests that the IVs are individually strong, the low R^2^ indicates that they do not capture much of the variance in the exposure.

## 5 Conclusion

This meta-analysis demonstrates that patients with NAFLD/MAFLD have an increased risk of colorectal polyps. However, the results of the MR analysis do not support a significant causal effect of NAFLD/MAFLD for colorectal polyps. Therefore, in light of the escalating prevalence of NAFLD and colorectal polyps, coupled with their significant health implications, there is an urgent need for studies with larger sample sizes and greater technical diversity in population cohorts.

## Data Availability

The original contributions presented in the study are included in the article/Supplementary Material, further inquiries can be directed to the corresponding author.
