# Supplementary material for: Nonalcoholic or metabolic-associated fatty liver disease and colorectal polyps: evidence from meta-analysis and two-sample Mendelian randomization
Source: Front Genet. 2024 Aug 9;15:1422827. doi: 10.3389/fgene.2024.1422827 (PMC11341362; doi:10.3389/fgene.2024.1422827)
Supplement: Supplementary file 1 [file Table1.DOCX]

**Table 1 Characteristics of studies on between NAFLD/MAFLD and the risk of colorectal polyps**

| **Author**  **(year)** | **country** | **Sex**  **(M/F)** | **Age (year)** | **Polyps in NAFLD/MAFLD** | **Polyps in control** | **Study type** | **Adjusted confounding factors** | **Type of polyps** |
| --- | --- | --- | --- | --- | --- | --- | --- | --- |
| Yang Y et al. (2023) | China | 1728/1300 | 54.4±14.0 | NAFLD (926) | Non-NAFLD (506) | Retrospective cross-sectional | age, sex, TG, TC, LDL-C and HDL-C. | Adenomatous |
| Touzin N T et al. (2011) | USA | 120/113 | 54.7±6.0 | NAFLD (23) | Non-NAFLD (35) | Retrospective cohort observational | race, BMI, and family history | Adenomas |
| Mahamid M et al. (2017) | Israel | 115/108 | 41.1±12.5 | NAFLD (14) | Non-NAFLD (16) | Retrospective cohort observational | gender, age, C-reactive protein and  smoking | hyperplastic polyp |
| Lesmana C R A et al. (2020) | Korea | 72/66 | 56.8±15.3 | NAFLD (30) | Non-NAFLD (19) | Retrospective | No adjusted | Adenoma |
| Hwang S T et al. (2010) | Korea | 1911/2917 | 47.0±9.0 | NAFLD (231)  MAFLD (67) | Non-NAFLD (325)  Non-MAFLD (258) | Cross-sectional | age, gender, smoking, NAFLD, metabolic syndrome, hypertension and diabetes | Adenomatous polyp |
| Cho Y et al. (2019) | China | 230/246 | 55.9±12.9 | NAFLD (133) | Non-NAFLD (20) | Cohort | Age, Sex, Diabetes mellitus, Antidiabetic drug  use, Hypertension, Antihypertensive  drug use, Statin use, Smoking, hsCRP, HOMA‐IR, Lobular inflammation, Ballooning, Steatosis grade, Significant fibrosis, Histological spectrum of NAFLD | Low‐grade tubular adenoid,  Advanced colorectal neoplasm |
| Chen Q F et al. (2017) | China | 2430/1256 | 47.5±10.6 | NAFLD (492) | Non-NAFLD (1479) | Retrospective cross-sectional | gender, age, smoking, alcohol and  MS. | Adenomatous polyps, Hyperplastic polyps |
| Chao G et al. (2020) | China | 469/249 | 49.4±8.2 | NAFLD (81) | Non-NAFLD (92) | Retrospective | No adjusted | Adenoma |
| Blackett J W et al. (2020) | USA | 183/186 | 60.4±8.9 | NAFLD (50) MAFLD (40) | Non-NAFLD (69)  Non-MAFLD (90) | Retrospective cross-sectional | Rates of hyper-lipidemia, diabetes, and obesity | Adenoma |
| Bhatt B D et al. (2015) | USA | 398/193 | 59.8 | NAFLD (40) | Non-NAFLD (208) | Retrospective cohort | age | Adenoma, Inflammatory, Hyperplastic, |
| Seo J Y et al. (2021) | Korea | 2130/1311 | 52.4±9.1 | NAFLD  (390) MAFLD (374) | Non-NAFLD (500)  Non-MAFLD (636) | Retrospective cohort | age, sex, smoking, triglyceride level, HDL cholesterol level, hypertension, visceral fat area, diabetes, and body mass index | Adenoma |
| Li Y et al. (2019) | China | 566/523 | 54.5±0.6 | NAFLD (142) | Non-NAFLD (125) | Retrospective cohort | sex, NAFLD, CAP, body mass index, triglyceride, aspartate aminotransferase, and fasting plasma glucose | Adenoma |
| Huang K W et al. (2013) | China | 890/612 | 53.7±9.7 | NAFLD (120) | Non-NAFLD (96) | Retrospective cohort | Age, Body mass index, Male gender, Nonalcoholic fatty liver disease, Smoking, Hypertension, Diabetes mellitus | Adenoma |
| Fukunaga S et al. (2021) | Japan | 100/24 | 59±11.1 | NAFLD (29) MAFLD (32) | Non-NAFLD (18) | Retrospective cross-sectional | No adjusted | Adenoma |
| Chang J et al. (2023) | Korean | 55788/92762 | 42.6±9.4 | MAFLD (   \| 11020 \| \| --- \|   ) | Non-MAFLD (14113) | cross- sectional | age, sex, centre, year of screening, smoking status, alcohol intake, educational level, a history of cardiovascular disease, and a family history of colorectal cancer. | Low- risk adenoma, or high- risk adenoma |
| Stadlmayr A. (2011) | Austria | 603/608 | 60.88±9.98 | NAFLD (215) | Non-NAFLD (126) | cohort | Sex, age, body mass index, Liver steatosis, Glucose intolerance | Tubular adenoma, advanced adenoma or carcinoma |
| Fliss-Isakov N. (2011) | Israel | 416/412 | 58.4±6 6.6 | NAFLD (162) | Non-NAFLD (260) | case-control | age, gender, BMI, low socioeconomic status, Ashkenazi origin, family history of colorectal malignancy, smoking (ever), alcohol consumption, physical inactivity, and use of statins, aspirin, and nonsteroidal anti-inflammatory drugs. | Hyperplastic polyps, Serrated adenomas, Adenomas |
