# Supplementary material for: Nonalcoholic or metabolic-associated fatty liver disease and colorectal polyps: evidence from meta-analysis and two-sample Mendelian randomization
Source: Front Genet. 2024 Aug 9;15:1422827. doi: 10.3389/fgene.2024.1422827 (PMC11341362; doi:10.3389/fgene.2024.1422827)
Supplement: Supplementary file 2 [file Table2.DOCX]

**Table 2 Quality assessment of included studies via the Newcastle-Ottawa scale (NOS).**

| **Study** | **Selection** | | | | **Comparability control for important factor** | **Exposure** | | | **Total score** |
| --- | --- | --- | --- | --- | --- | --- | --- | --- | --- |
|  | **Adequate case definition** | **Representativeness**  **Of the cases** | **Selection of**  **controls** | **Deﬁnition of**  **controls** |  | **Ascertainment**  **of exposure** | **Same method of ascertainment for cases and controls** | **Non-response rate** |  |
| Yang Y et al. (2023) | 1 | 1 | 1 | 0 | 2 | 1 | 1 | 0 | 7 |
| Touzin N T et al. (2011) | 1 | 1 | 1 | 1 | 2 | 1 | 1 | 0 | 8 |
| Mahamid M et al. (2017) | 1 | 1 | 1 | 1 | 2 | 1 | 1 | 0 | 8 |
| Lesmana C R A et al. (2020) | 1 | 1 | 1 | 1 | 1 | 1 | 1 | 0 | 7 |
| Hwang S T et al. (2010) | 1 | 1 | 1 | 0 | 2 | 1 | 1 | 0 | 7 |
| Cho Y et al. (2019) | 1 | 1 | 1 | 1 | 2 | 1 | 1 | 0 | 8 |
| Chen Q F et al. (2017) | 1 | 1 | 1 | 1 | 2 | 1 | 1 | 0 | 8 |
| Chao G et al. (2020) | 1 | 1 | 1 | 1 | 2 | 1 | 1 | 0 | 8 |
| Blackett J W et al. (2020) | 1 | 1 | 11 | 1 | 2 | 1 | 1 | 0 | 8 |
| Bhatt B D et al. (2015) | 1 | 1 | 1 | 1 | 2 | 1 | 1 | 0 | 8 |
| Seo J Y et al. (2021) | 1 | 1 | 1 | 1 | 2 | 1 | 1 | 0 | 8 |
| Li Y et al. (2019) | 1 | 1 | 1 | 1 | 2 | 1 | 1 | 0 | 8 |
| Huang K W et al. (2013) | 1 | 1 | 1 | 1 | 2 | 1 | 1 | 0 | 8 |
| Fukunaga S et al. (2021) | 1 | 1 | 1 | 1 | 1 | 1 | 1 | 0 | 7 |
| Chang J et al. (2023) | 1 | 1 | 1 | 1 | 2 | 1 | 1 | 0 | 8 |
| Stadlmayr A. (2011) | 1 | 1 | 1 | 1 | 2 | 1 | 1 | 0 | 8 |
| Fliss-Isakov N. (2011) | 1 | 1 | 1 | 1 | 2 | 1 | 1 | 0 | 8 |
