# Supplementary material for: Nonalcoholic or metabolic-associated fatty liver disease and colorectal polyps: evidence from meta-analysis and two-sample Mendelian randomization
Source: Front Genet. 2024 Aug 9;15:1422827. doi: 10.3389/fgene.2024.1422827 (PMC11341362; doi:10.3389/fgene.2024.1422827)
Supplement: Supplementary file 3 [file Table3.DOCX]

**Table3 Subgroup analysis of the incidence of colorectal polyps with NAFLD/MAFLD**

| Subgroup | Study included | OR (95%CI) | P^(interaction)^ |
| --- | --- | --- | --- |
| **NAFLD** |  |  |  |
| Gender |  |  | 0.46 |
| Male | 3 | 1.63(1.11-2.38) |  |
| Female | 3 | 1.39(1.15-1.67) |  |
| Age |  |  | 0.15 |
| ＜50 year | 4 | 1.41(1.17-1.69) |  |
| ≥ 50 year | 10 | 1.64(1.48-1.82) |  |
| Region |  |  | 0.19 |
| Asia | 12 | 1.53(1.43-1.64) |  |
| Non-Asia | 4 | 1.75(1.45-2.13) |  |
| **MAFLD** |  |  |  |
| Age |  |  | 0.05 |
| ＜50 year | 2 | 2.06(1.47-2.88) |  |
| ≥ 50 year | 3 | 1.47(1.35-1.59) |  |
| Diagnosis deference |  |  | 0.73 |
| NAFLD-MAFLD | 2 | 1.70(1.42-2.03) |  |
| MAFLD | 3 | 1.75(1.71-1.80) |  |

NAFLD: nonalcoholic fatty liver disease; MAFLD: Metabolic-Associated Fatty Liver Disease; 2-DM: Type 2 diabetes mellitus.
