# Supplementary material for: Nonalcoholic or metabolic-associated fatty liver disease and colorectal polyps: evidence from meta-analysis and two-sample Mendelian randomization
Source: Front Genet. 2024 Aug 9;15:1422827. doi: 10.3389/fgene.2024.1422827 (PMC11341362; doi:10.3389/fgene.2024.1422827)
Supplement: Supplementary file 4 [file Table4.DOCX]

**Table 4**: Associations of genetic instruments for NAFLD with colorectal polyps

|  |  |  |  | Exposure (NAFLD/MAFLD) | | |  | Outcome (Colorectal polyps) | | |
| --- | --- | --- | --- | --- | --- | --- | --- | --- | --- | --- |
| Instrumental SNP | Effect allele | Other allele |  | β | SE | P value |  | β | SE | P value |
| rs1260326 | C | T |  | -0.136025 | 0.0208660 | 2.53630e-11 |  | -7.25233e-05 | 0.000214625 | 0.7400000 |
| rs17321515 | G | A |  | -0.154093 | 0.0207828 | 1.81343e-13 |  | -1.27993e-04 | 0.000210322 | 0.5400000 |
| rs2642442 | T | C |  | 0.137690 | 0.0227583 | 7.67132e-10 |  | -1.79940e-04 | 0.000225517 | 0.4200000 |
| rs3747207 | A | G |  | 0.369714 | 0.0229366 | 6.74062e-60 |  | 2.23223e-04 | 0.000255524 | 0.3800000 |
| rs429358 | C | T |  | -0.199223 | 0.0304411 | 2.16920e-11 |  | 0 6.27212e-04 | 0.000291014 | 0.0309999 |
| rs73001065 | C | G |  | 0.345021 | 0.0348771 | 1.08143e-24 |  | -3.13664e-04 | 0.000378865 | 0.4100000 |

***P* value < 5×10^-8^ for reporting genome-wide significance. EAF: effect allele frequency. SE: standard error; SNP,: single nucleotide polymorphism. NAFLD: non-alcoholic fatty liver disease. MAFLD: Metabolic-Associated Fatty Liver Disease.**
